# Sequential Targeting of PLK1 and PARP1 Reverses the Resistance to PARP Inhibitors and Enhances Platin-Based Chemotherapy in BRCA-Deficient High-Grade Serous Ovarian Cancer with KRAS Amplification

**DOI:** 10.3390/ijms231810892

**Published:** 2022-09-17

**Authors:** Khayal Gasimli, Monika Raab, Morva Tahmasbi Rad, Elisabeth Kurunci-Csacsko, Sven Becker, Klaus Strebhardt, Mourad Sanhaji

**Affiliations:** 1 Department of Gynecology, University Hospital, 60590 Frankfurt am Main, Germany; 2 German Cancer Consortium (DKTK), German Cancer Research Center, 69120 Heidelberg, Germany

**Keywords:** high-grade serous ovarian cancer, BRCA2 deficiency, KRAS amplification, DNA damage, PARP inhibitor resistance, PLK1-based combinatorial therapy

## Abstract

Ovarian cancer (OC) accounts for approximately 4% of cancer deaths in women worldwide and is the deadliest gynecologic malignancy. High-grade serous ovarian cancer (HGSOC) is the most predominant ovarian cancer, in which BRCA1/2 gene mutation ranges from 3 to 27%. PARP inhibitors (PARPi) have shown promising results as a synthetically lethal therapeutic approach for BRCA mutant and recurrent OC in clinical use. However, emerging data indicate that BRCA-deficient cancers may be resistant to PARPi, and the mechanisms of this resistance remain elusive. We found that amplification of KRAS likely underlies PARPi resistance in BRCA2-deficient HGSOC. Our data suggest that PLK1 inhibition restores sensitivity to PARPi in HGSOC with KRAS amplification. The sequential combination of PLK1 inhibitor (PLK1i) and PARPi drastically reduces HGSOC cell survival and increases apoptosis. Furthermore, we were able to show that a sequential combination of PLK1i and PARPi enhanced the cellular apoptotic response to carboplatin-based chemotherapy in KRAS-amplified resistant HGSOC cells and 3D spheroids derived from recurrent ovarian cancer patients. Our results shed new light on the critical role of PLK1 in reversing PARPi resistance in KRAS-amplified HGSOC, and offer a new therapeutic strategy for this class of ovarian cancer patients where only limited options currently exist.

## 1. Introduction

Ovarian cancer (OC) is the seventh most frequently diagnosed and eighth leading cause of cancer death among women worldwide [1,2]. The most prevalent form of ovarian cancer is high-grade serous ovarian carcinoma (HGSOC), often diagnosed at an advanced stage and characterized by a poor overall survival rate. The current standard therapy comprises extensive cytoreductive surgery followed by systemic chemotherapy with Carboplatin and Paclitaxel [3,4]. Based on recent morphological, immunohistochemical, and molecular genetic studies, a two-tiered carcinogenesis model divides epithelial ovarian cancer into two categories, type I and type II [5]. Type I contains low-grade serous carcinomas, low-grade endometrioid carcinomas, clear cell carcinomas, and mucinous carcinomas. This type is driven by oncogenic KRAS and displays a variety of somatic mutations in KRAS, BRAF, PTEN, PIK3CA, CTNNB1, and ARID1A, and very seldomly in P53 [6,7]. Type II encompasses high-grade serous, high-grade endometrioid, and malignant mixed mesodermal tumors [6]. While it rarely contains KRAS mutations, it shows RAS pathway activation in about 25% of tumors [8,9] and KRAS amplification in 13.9% of patients, especially in HGSOC patients, who represent the prototypical type II ovarian cancer [5,9]. About 50% of HGSOC patients show homologous recombination (HR) repair pathway deficiency, of which almost 30% are due to somatic/germline or epigenetic loss of BRCA1/2 [9,10,11]. HR deficiency and loss of BRCA1/2 likely contribute to the efficacy of platinum and poly (adenosine diphosphate-ribose) polymerase inhibitor (PARPi) treatments. PARP is a crucial component of single-strand break (SSB) repair. Upon recognizing SSBs, PARP activates the base excision repair pathway [12,13], restoring the DNA defects. Alternatively, when double-strand breaks (DSBs) are already occurring, PARP is involved in the repair by recruiting the repair protein of the HR or the non-homologous end joining (NHEJ) pathways [14]. Several PARPis have been developed and approved by the FDA and the European Medicine Agency for patient use, including Olaparib, Rucaparib, and Niraparib [15,16]. Unfortunately, PARP inhibition has shown only a temporary benefit for patients who start to develop resistance. Therefore, it is vital to propose new therapeutic combinations to palliate resistance to PARPi, and especially to study the molecular origins behind this resistance.

The serine/threonine Polo-Like Kinase 1 (PLK1) performs important roles in regulating cell division. PLK1 regulates cytokinesis and controls mitotic entry, spindle assembly, and chromosome dynamics [17,18,19,20,21]. In addition, PLK1 is implicated in the modulation of DNA damage repair through its active regulation of several essential repair proteins involved in HR, such as Rad51 and Mre11 [22,23]. More importantly, PLK1 activity is necessary for the cell to resume the cell cycle after recovery from DNA damage-induced G2 arrest [24,25]. However, PLK1 misregulation has been described in different tumor types, contributing to tumor development and progression [26,27], and it is reported to be overexpressed in many cancers compared to the normal tissue [26]. Furthermore, PLK1 expression has prognostic relevance in ovarian cancer [28,29], and its overexpression is associated with poor patient outcomes [30]. While studies have indicated that the use of PLK1 inhibition may overcome drug resistance in chemotherapy [31,32,33], other research has aimed to enhance the efficacy of standard therapy through PLK1i-based combinations, such as in ovarian cancer with CCNE1 amplification, where the PLK1 inhibitor BI6727 in combination can potentiate the effect of paclitaxel and boost the efficacy of cisplatin in cervical cancer [34,35].

We found that the expression of KRAS in BRCA2-deficient HGSOC may trigger an adaptive response that mediates resistance to PARPi. Furthermore, we identified that PLK1i reverses PARP resistance of HR-deficient HGSOC with KRAS amplification. Consistent with this, the sequential combination of PARPi and PLK1i enhances the cellular apoptotic response and strongly reduces the cell survival of BRCA2-deficient HGSOC with and without KRAS amplification. Moreover, our data show that PLK1i and PARPi increase the efficacy and boost the response to Carboplatin-based chemotherapy in BRCA2-deficient HGSOC cells and patient tumor-derived 3D-spheroids independent of KRAS status.

## 2. Results

### 2.1. KRAS Gene Expression and Survival of Ovarian Cancer Patients

The KM plotter tool was used [36] to explore the prognostic value of KRAS expression in ovarian cancer patients and to establish a correlation between gene expression and patient survival. We performed the analysis on 943 patients with HGSOC, predominantly classified as stage III (FIGO) [37], and who received Platinum-containing therapy. Of the patients, 56% showed low and 53% high KRAS expression (Supplementary Figure S1A). Analysis indicates that increased KRAS gene expression significantly reduces overall survival (OS) from 49 to 39.87 months (*p* = 0.0038) (Supplementary Figure S1A). These data imply that KRAS gene expression may predict poor prognosis in HGSOC patients and is indicative of a modest response to platinum-based chemotherapy.

### 2.2. Evaluation of the Cytotoxic Effects of Olaparib and BI6727 on BRCA2-Deficient HGSOC Cells with and without KRAS Amplification

Numerous studies have reported the development of synthetic lethality when BRCA1/2-deficient tumors are treated with PARP inhibitors [16,38,39]. However, the response to PARPi is frequently transient, and many patients who initially respond to PARPi become resistant, leading to a limited overall survival benefit [40]. Intriguingly, reports link PARPi resistance with elevated RAS/MAPK signaling [41,42]. Considering the co-dependency between PLK1 and KRAS mutant tumors [43,44], we sought to investigate whether PLK1 inhibition sensitizes HGSOC with KRAS amplification to PARPi. We selected two BRCA2-deficient HGSOC cell lines for this study, OVSAHO and KURAMOCHI, the latter of which harbors KRAS and MYC amplification (Supplementary Figure S1B) [45,46]. We evaluated the expression of PARP, PLK1, and KRAS in a small spectrum of asynchronized HGSOC cell lines using western blot (Supplementary Figure S1C,D). KURAMOCHI and OVSAHO both express high levels of PLK1 (Supplementary Figure S1C). However, in contrast to OVSAHO, KURAMOCHI cells show decreased expression of PARP, suggesting low sensitivity of KURAMOCHI cells to PARP inhibition (Supplementary Figure S1C). More interestingly, we found high expression of KRAS in KURAMOCHI cells compared to OVSAHO or OVCAR-8, and these increased KRAS levels were associated with upregulation of the RAF/MEK/ERK pathway, as indicated by the high levels of pERK1/2 (Supplementary Figure S1D,E).

**Figure 1:**
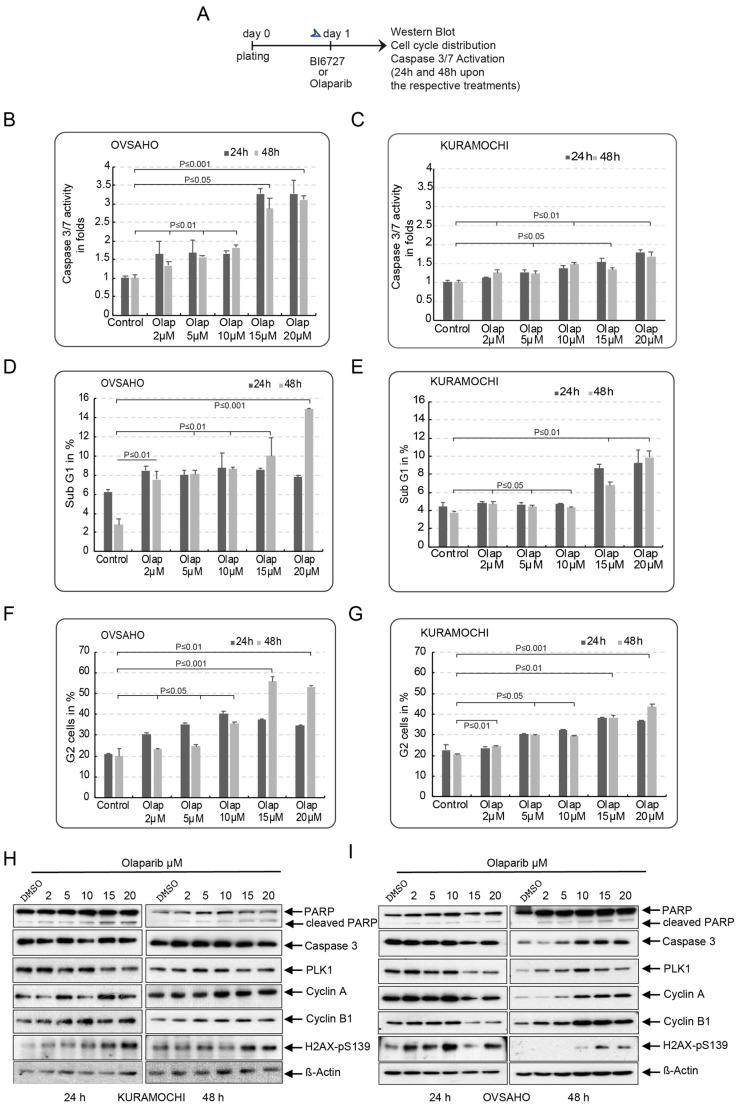
KRAS amplification mediates resistance to PARPi in HGSOC cells with BRCA2 deficiency. (**A**)—Treatment schedule: KURAMOCHI and OVSAHO cells were treated with increasing concentrations of the PARP inhibitor Olaparib, 2 µM to 20 µM. Cells were harvested after 24 h and 48 h, and further experiments were carried out. (**B**,**C**) Apoptosis was then assessed at the indicated time points by measuring Caspase 3/7 activity in the lysates of cells. The results are presented as mean ± SD. (*n* = 3). (**D**,**E**) Cell death was assessed by quantifying the sub-G1 phase. The results are presented as mean ± SD. (*n* = 3). (**F**,**G**) The cell cycle distribution of treated KURAMOCHI and OVSAHO was analyzed 24 h and 48 h post-Olaparib treatment using FACS. The resulting G2 fractions of both cell lines are displayed. The results are presented as mean ± SD. (*n* = 3). (**H**,**I**) OVSAHO and KURAMOCHI cell lysates treated as in (**A**) were prepared for Western blot analysis after 24 h and 48 h using the indicated antibodies. The results of the (**B**–**G**) are presented as mean ± SD. (*n* = 3, *p* ≤ 0.001, *p* ≤ 0.01, *p* ≤ 0.05).

To evaluate the single effect of Olaparib and BI6727 on the BRCA2-deficient HGSOC cell lines KURAMOCHI (with KRAS amplification) and OVSAHO, we treated both cell lines with increasing concentrations of Olaparib (2–20 µM) and BI6727 (10–100 nM) for 24 h and 48 h. Apoptosis was assessed by measuring the activation of caspase 3/7 (Figure 1A–C). Both cell lines showed a modest increase in apoptosis at low concentrations of Olaparib (2–10 µM). However, OVSAHO cells appeared to be slightly more sensitive to Olaparib, with an increase of 1.8-fold caspase 3/7 activation for OVSAHO after 48 h at 10 µM Olaparib, and 1.5-fold for KURAMOCHI vs. control cells (Figure 1B,C). More intriguingly, at higher concentrations (15 µM and 20 µM), KURAMOCHI cells showed higher resistance to Olaparib than OVSAHO, confirming the results of a previous study [47]. While 20 µM Olaparib only increased caspase 3/7 activation 1.7-fold in KURAMOCHI cells after 48 h, OVSAHO showed a substantial increase in caspase 3/7 activation by 3.1-fold compared to untreated cells (Figure 1B,C). Similarly, using flow cytometry to evaluate the apoptotic sub-G1 cell fraction revealed that OVSAHO cells are more sensitive to PARP inhibition than KURAMOCHI cells at low and high concentrations, with 4.4% vs. 8.6% at 10 µM Olaparib and 8.6% vs. 15% at 20 µM Olaparib, respectively, for KURAMOCHI and OVSAHO after 48 h (Figure 1D,E and Figure S2A,B). Cell cycle analysis showed that Olaparib caused a dose-dependent increase in the G2/M fraction of KURAMOCHI and OVSAHO cells after 48 h, suggesting that Olaparib can accumulate in both cell lines at the G2/M phase (Figure 1F,G and Figure S2A,B). The increased expression of Cyclin A and Cyclin B1 48 h post-treatment confirmed that Olaparib induces G2/M arrest in both cell lines (Figure 1H,I). Furthermore, 48 h treatment of both cell lines with 15 µM and 20 µM Olaparib triggers the formation of γ-H2AX foci, indicating the occurrence of double-strand breaks in the DNA (Figure 1H,I).

We further assessed the apoptotic potential of PLK1 inhibition on both cell lines. To this end, we incubated KURAMOCHI and OVSAHO cell lines with increasing doses of the specific PLK1 inhibitor BI6727 (10–100 nM) for 24 h and 48 h (Supplementary Figure S3A). While treatment with low doses of BI6727 (up to 20 nM) showed a very weak apoptotic response in OVSAHO cells (Supplementary Figure S3B), the KURAMOCHI cells containing KRAS amplification displayed high sensitivity to PLK1 inhibition (Supplementary Figure S3C). Thus, Caspase 3/7 activation rose 2.2- and 3.2-fold for 10 nM and 20 nM BI6727, respectively, in KURAMOCHI cells (Supplementary Figure S3C). Although high concentrations of BI6727 stimulated the apoptotic response in both cell lines, the response of KURAMOCHI remained higher than that of OVSAHO (Supplementary Figure S3B,C). At a concentration of 100 nM, BI6727 induced a 6.6-fold increase in Caspase 3/7 activation in KURAMOCHI 24 h after treatment, compared to only 2.2-fold in OVSAHO. Upon 48 h, these values changed to 5.8-fold compared to 4.4-fold in KURAMOCHI and OVSAHO, respectively (Supplementary Figure S3B,C). Furthermore, FACS analysis of the sub-G1 distribution in both cell lines confirmed the sensitivity of KURAMOCHI to BI6727 treatment compared with OVSAHO cells (Supplementary Figure S3D,E). Thus, increasing concentrations of BI6727 caused a more substantial increase in the sub-G1 fraction of KURAMOCHI cells; 50 nM and 100 nM BI6727 respectively triggered 33% and 37% apoptosis in KURAMOCHI cells, whereas OVSAHO showed only 14% and 21% cell death, respectively, after 48 h (Supplementary Figure S3D,E). Conversely, BI6727 treatment induced a similar G2/M arrest in both cell lines, as indicated by the comparable dose-dependent increase in the G2/M distribution (Supplementary Figure S3F,G).

These data suggest that KRAS amplification renders BRCA2-deficient HGSOC cells insensitive to Olaparib treatment. Interestingly, KURAMOCHI cells with KRAS amplification showed better apoptotic response to PLK1i as a single agent. Based on these observations, we hypothesized that PLK1 inhibition would restore the antineoplastic activity and potentiate Olaparib-associated cell death in BRCA2-deficient and KRAS-amplified HGSOCs.

### 2.3. Sequential Inhibition of PLK1 and PARP Sensitizes HGSOC Cells to Olaparib

**Figure 2:**
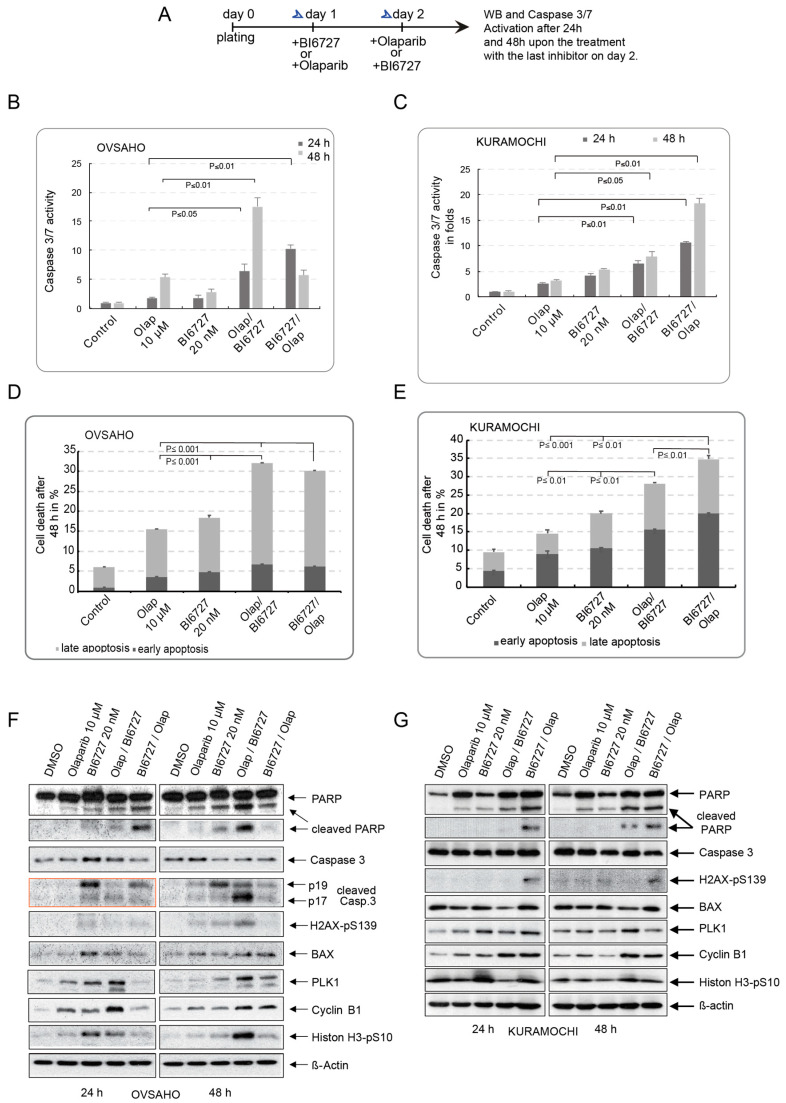
Sequential PARPi and PLKi sensitize BRCA2-deficient HGSOC to Olaparib treatment independent of the KRAS status. (**A**) Treatment schedule: KURAMOCHI and OVSAHO cells were first incubated with a single treatment consisting of Olaparib (10 µM) or BI67272 (20 nM) and the sequential combinations Olaparib/BI6727 or BI6727/Olaparib at days 1 and 2. The cells were harvested 24 h and 48 h post-treatments, and further experiments were carried out. (**B**,**C**) Apoptosis was assessed by measuring Caspase 3/7 activity in cell lysates of cells incubated with the different single or combinatorial treatments. The results are presented as mean ± SD. (*n* = 3, *p* ≤ 0.001, *p* ≤ 0.01, *p* ≤ 0.05). (**D**,**E**) Cell death was then assessed after 24 h and 48 h using Annexin V/AAD. The results are presented as mean ± SD. The total apoptosis (early and late) was used for statistical analysis. (*n* = 3, *p* ≤ 0.001, *p* ≤ 0.01). (**F**,**G**) The cell lysates of KURAMOCHI and OVSAHO cells treated with the single and the combination treatments were prepared for Western Blot with the indicated antibodies.

To explore the potential of PLK1 inhibition in sensitizing KURAMOCHI cells with KRAS amplification and OVSAHO cells to PARP inhibition, we assessed the effects of the combinatorial therapy based on PARPi and PLK1i. To do this, KURAMOCHI and OVSAHO cells underwent a single treatment using BI6727 (20 nM) or Olaparib (10 µM) and a combinatorial treatment based on a sequential treatment with Olaparib on day one followed by BI6727 on day two, or vice versa (Figure 2A). Cells were harvested after 24 h and 48 h, and apoptosis was first measured by Caspase 3/7 activation. The single incubation of KURAMOCHI and OVSAHO cells with 10 µM Olaparib increased the incidence of apoptosis at all indicated time points compared to controls (Figure 2B,C). However, OVSAHO showed higher Olaparib-triggered activation of Caspase 3/7 than in KURAMOCHI cells, particularly after 48 h treatment (5.3-fold vs. 3.1-fold, respectively, for OVSAHO and KURAMOCHI) (Figure 2B,C). Furthermore, and in line with the previous result, single BI6727 treatment (20 nM) showed only moderate apoptosis induction in OVSAHO cells compared with KURAMOCHI at all time points (1.8-fold vs. 4.1-fold after 24 h and 2.8-fold vs. 5.4-fold after 48 h, respectively, for OVSAHO and KURAMOCHI) (Figure 2B,C). This confirms that, in contrast to OVSAHO, KURAMOCHI cells with KRAS amplification show less sensitivity to PARPi and a higher apoptotic response to PLK1 inhibition.

Prior to sequential treatment on OVSAHO and KURAMOCHI, we compared concurrent addition of PARPi and PLK1i with sequential BI6727/Olaparib and Olaparib/BI6727 therapy. We found that simultaneous addition of PLK1i and PARPi elicited a similar response to sequential addition of BI6727 followed by Olaparib (data not shown). Therefore, we decided to pursue both sequential treatments, Olaparib/BI6727 and BI6727/Olaparib, which showed different results.

For the double combinations, we observed that either sequential combination, Olaparib/BI6727 or BI6727/Olaparib, could trigger a significant increase in apoptosis compared with the effect of the single treatments (Figure 2B,C). Nevertheless, we observed that the two cell lines responded differently depending on the addition sequence of both compounds. While the combination of Olaparib/BI6727 in OVSAHO cells triggered a time-dependent increase in apoptosis, from 6.4-fold after 24 h to 17.6-fold after 48 h treatment, it was only augmented to 6.4-fold and 7.8-fold after 24 h and 48 h in KURAMOCHI cells (Figure 2B,C). Intriguingly, the outcome changed as KURAMOCHI were treated with the combination BI6727/Olaparib. This combination showed greater efficacy on KURAMOCHI cells apoptotic response. Thus, after 24 h, caspase 3/7 activation increased by 10.5-fold, and after 48 h, an upsurge of 17.8-fold was observed (Figure 2C). However, this robust apoptotic response could not be elicited in OVSAHO cells after sequential BI6727/Olaparib treatment. Instead, upon reaching a maximum apoptotic response after 24 h (10.3-fold), this induction dropped to values similar to single agent treatment after 48 h (Figure 2B). This could be explained, at least in part, by the fact that this combination does not seem to be able to maintain the formation of DSB sites in OVSAHO cells 48 h after treatment, as evidenced by the weak H2AX-pS139 signal (Figure 2F), thus impairing the intracellular apoptotic response of OVSAHOs to BI6727/Olaparib treatment after 48 h (Figure 2B).

We further investigated the cell death induced by the two sequential combinations using the Annexin V/7-AAD assay after 48 h incubation. Here, we observed that, regardless of the sequence in which Olaparib and BI6727 were added to OVSAHO or KURAMOCHI, the double combination significantly boosted the cell death in contrast to single treatments, indicating that PLK1 indeed sensitizes BRCA2-deficient HGSOC cells to Olaparib treatment (Figure 2D,E and Figure S4A,B). However, KURAMOCHI cells, which contain KRAS amplification, again showed a preferential and better apoptotic response when cells were treated first with BI6727 and then with Olaparib (28% apoptosis for the Olaparib/BI6727 sequence versus 34.8% for the BI6727/Olaparib combination) (Figure 2E and Figure S4B). Finally, Western Blot analysis confirmed that sequential treatment with Olaparib/BI6727 was the most efficient combination for OVSAHO cells, as indicated by the increased levels of the apoptosis markers cleaved Caspase 3 and BAX, which was concomitant with an accumulation of the DSB marker H2AX-pS139 (γ-H2AX) (Figure 2F,G). Together, these data argue that PLK1 inhibition boosts the effect of PARPi in BRCA2-deficient HGSOC cells and reverses the resistance of BRCA2-deficient HGSOC with KRAS amplification to PARP inhibition.

**Figure 3:**
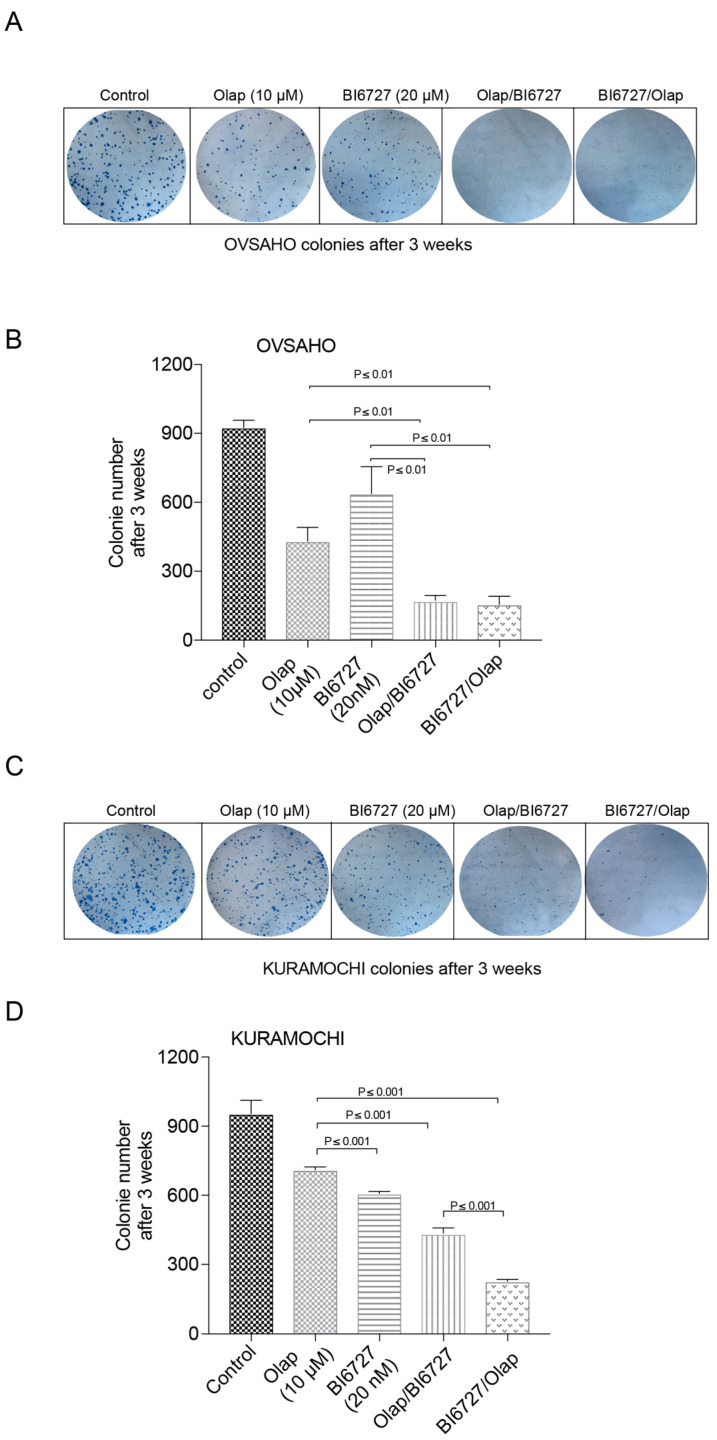
Sequential PARPi and PLKi treatment reduced the 2D clonogenic potential of HR-deficient HGSOC. (**A**,**C**) OVSAHO and KURAMOCHI cells grown in colonies were subjected to Coomassie blue staining. (**B**,**D**) The number of colonies was counted and is represented as a bar graph. The results are presented as mean ± SD. (*n* = 3, *p* ≤ 0.001, *p* ≤ 0.01).

To study the impact of PARP and PLK1 inhibitions on the long-term clonogenic potential of OVSAHO and KURAMOCHI cells, we tested the cell’s ability over three weeks to form 2D colonies upon treatment with single and combinatorial treatments (Figure 3). First, we observed that single PARP inhibition showed moderate efficacy in KURAMOCHI in terms of limiting clonogenic potential compared to that obtained in the OVSAHO cell line (430 vs. 710 colonies for OVSAHO and KURAMOCHI, respectively), again suggesting that KRAS amplification desensitizes KURAMOCHI cells to PARP inhibition (Figure 3B,D). In line with the above-described results, the inhibition of PARP and PLK1 markedly reduced the clonogenic potential in both HR-deficient HGSOC cell lines irrespective of treatment sequence. Indeed, both combinations were similarly and significantly able to reduce OVSAHO clonogenic ability compared to single treatments (Figure 3A,B). Interestingly, in the case of the KURAMOCHI cells, the sequential BI6727/Olap treatment showed a much stronger inhibitory effect on colony formation compared to Olap/BI6727 combination or single treatments (Figure 3C,D). These data reiterate that PLK1 inhibition improves the PARP-based treatment of HR deficient HGSOC. Furthermore, sequential treatment with an initial BI6727 treatment followed by Olaparib significantly worsens the survival potential of the HR-deficient and KRAS-amplified KURAMOCHI cells.

### 2.4. PLK1 and PAPR1 Inhibitions Lead to the Persistence of DSBs and Reduce the Survival of BRCA2-Defective HGSOC Cell Lines over the Long Term

**Figure 4:**
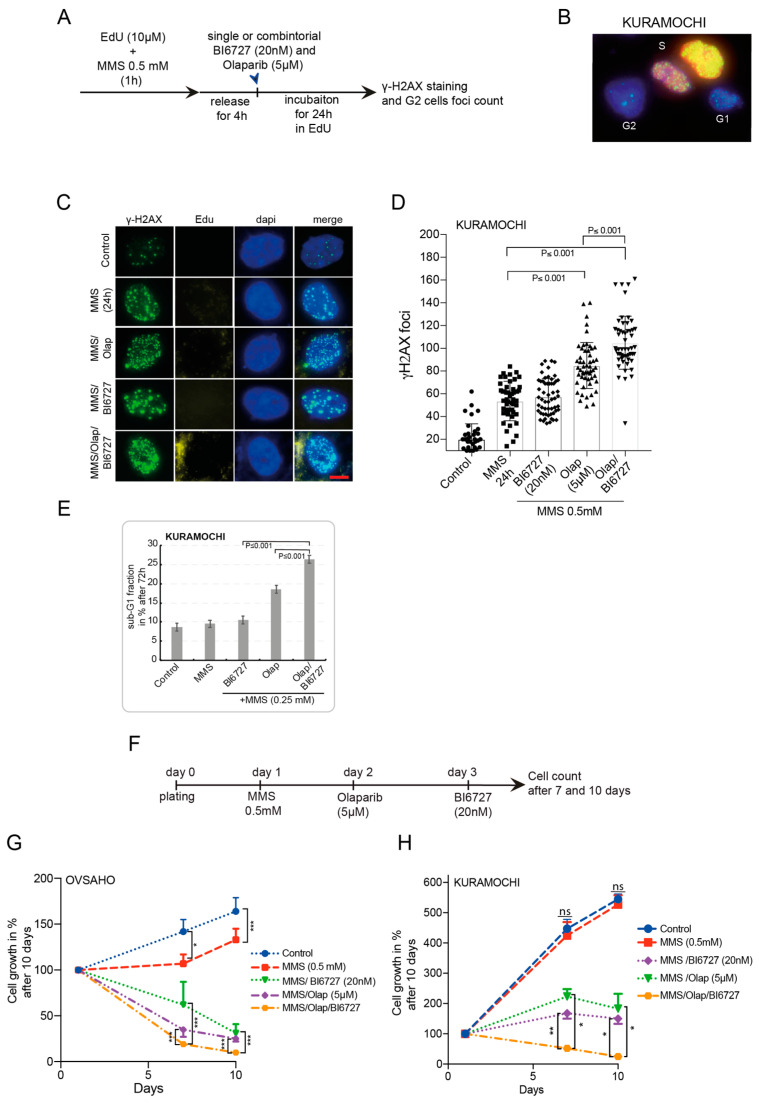
Combining PLK1i and PARPi enhances the DNA damage effect of MMS and reduces the viability of BRCA2-deficient HGSOC with KRAS amplification. (**A**)—Treatment schedule: KURAMOCHI cells were EdU labeled and pulse-treated with MMS (0.5 mM) for 1 h. The cells were released in EdU-containing medium for 4 h, then treated with the single agents or combined for 24 h in the presence of EdU. γ-H2AX foci were analyzed in EdU-negative G2 phase cells. (**B**) EdU-positive cells were identified as S phase cells, whereas EdU-negative cells were classified as G1 (small DNA content) or G2 (large DNA content). (**C**) IF images show KURAMOCHI cells with γ-H2AX foci and negative EdU staining at 24 h post-treatment. (**D**) Quantification of γ-H2AX foci 24 h post-treatment. The results are presented as mean ± SEM (*n* = 50 cells per treatment, *p* ≤ 0.001). (**E**) Cell death was assessed in treated KURAMOCHI cells as in (**A**) by quantifying the sub-G1 phase after 72 h. The results are presented as mean ± SD. (*n* = 3, *p* ≤ 0.001). (**F**) Treatment schedule: KURAMOCHI and OVSAHO cells were pulse-treated with MMS (0.5 mM) for 1 h on day 1. Following this, cells were sequentially treated with Olaparib (5 µM) on day 2 and BI6727 (20 nM) on day 3. Cells were harvested and counted 7 and 9 days upon the completion of the combination treatment. (**G**,**H**) Growth kinetics of KURAMOCHI and OVSAHO cells treated with the different single agents and in combinations over 7 and 9 days. The results are presented as mean ± SD. (*n* = 3, *** *p* ≤ 0.001, ** *p* ≤ 0.01, * *p* ≤ 0.05).

Although PLK1 is implicated in the G2–M transition, it is not considered an essential kinase for mitosis entry during unperturbed cell division [48,49]. However, when cells are challenged with genotoxic stress in the form of double-strand breaks (DSB) during the G2 phase, cells fail to arrest if PLK1 is not efficiently inhibited, indicating the critical role of PLK1 for proper checkpoint recovery [24] and the adaptive response to DNA damage checkpoint [25]. Accordingly, we investigated whether PLK1, which is overexpressed in ovarian cancer [28,50], confers an adaptive response to HGSOC cells against DNA damaging agents and PARP inhibition. Therefore, we aimed to characterize the DSB repair ability of OVSAHO and KURAMOCHI cells after pulse treatment with the alkylating agent methyl methanesulfonate (MMS) [51] for 1 h to generate DSBs. Single Olaparib or BI6727 or both treatments in combination were added subsequently, and we quantified the number of γ-H2AX (H2AX-pS139) foci as a marker of DSBs after 24 h (Figure 4A and Figure S5A). To precisely analyze the cells retained in the G2 phase, we labeled the cells with the thymidine analog EdU (10 µM). EdU-positive cells were identified as S-phase cells, while the EdU-negative cells were classified as either G1 or G2 based on their DNA content (Figure 4B) [52]. Interestingly, by comparing the baseline level of γ-H2AX foci prior to treatment with MMS, we found that untreated KURAMOCHI cells show a higher number of spontaneous γ-H2AX foci (20 foci) compared to OVSAHO cells (10 foci) (Figure 4C,D and Figure S5B,C). This difference might be relevant, as high baseline DNA damage results in poor clinical outcomes upon radiotherapy and correlate with a weaker repair response following irradiation [53]. Furthermore, in contrast to the combination MMS/BI6727, which only marginally increased the effect of MMS in both cell lines, the inhibition of PARP following MMS treatment significantly increased γ-H2AX foci, and to an even greater extent in KURAMOCHI cells after 24 h incubation (Figure 4C,D and Figure S5B,C). More importantly, combining PARP and PLK1 inhibition after MMS treatment further increased the number of γ-H2AX foci and their intensity, indicating the accumulation and solid persistence of double-strand breaks and unrepaired DNA in G2 cells after 24 h. Thus, on average, we counted 66 foci of γ-H2AX in OVSAHO G2 cells for the combination MMS/Olap/BI6727 versus 105 foci in KURAMOCHI G2 cells (Figure 4C,D and Figure S5B,C).

Evidence suggests that tumor cells exposed to DSB-inducing agents are more likely to die within 24 h of treatment if they retain microscopically visible γ-H2AX foci [54]. In agreement with this, we observed that the most significant increase in the dead fraction of both cell lines (sub-G1) was visible up to 72 h post-MMS treatment combined with PARPi and PLK1i compared to the double combination MMS/Olap or MMS/BI6727 (Figure 4E and Figure S5D). Taken together, the combination of PARPi and PLK1i sensitizes HGSOC cells to DNA damage agents irrespective of the presence of KRAS amplification, eventually leading to cell death.

We further tested the efficacy of combining MMS with PARP and PLK1 inhibitions on the survival and growth of OVSAHO and KURAMOCHI cell lines over 10 days (Figure 4F–H). First, we observed that treatment with MMS (0.5 mM) did not affect the growth of KURAMOCHI when compared with untreated control cells. In contrast, OVSAHO cells showed a significant reduction in growth following comparable treatment (Figure 4G,H). This suggests that KRAS amplification and the high-baseline DSB observed in the previous experiment reduce the sensitivity of KURAMOCHI cells to DNA damage agents. Moreover, in OVSAHO cells, all combinations were efficient in blocking cellular growth and even reducing cell viability. Notably, the triple MMS/Olap/BI6727 was able to reduce the viability of OVSAHO cells to 10% after 10 days compared to 31% and 25% for MMS/BI6727 and MMS/Olap (Figure 4G). For the more resistant KURAMOCHI cells, the combination MMS/Olap/BI6727 was able to block the cell growth efficiently and significantly reduced cell survival, resulting in only 25% of cells left after 10 days of treatment, compared with 150% and 183% for MMS/BI6727 and MMS/Olap respectively (Figure 4H). These data show that combining the DNA damage agent with PLK1i and PARPi enhances the apoptotic response of the BRCA2-deficient OVSAHO cell line to DNA damage and restores the sensitivity of BRCA2-deficient and KRAS-amplified KURAMOCHI to this treatment.

### 2.5. Combined PARP and PLK1 Inhibition Mediates Sensitivity to CARBOPLATIN-Based Chemotherapy

**Figure 5:**
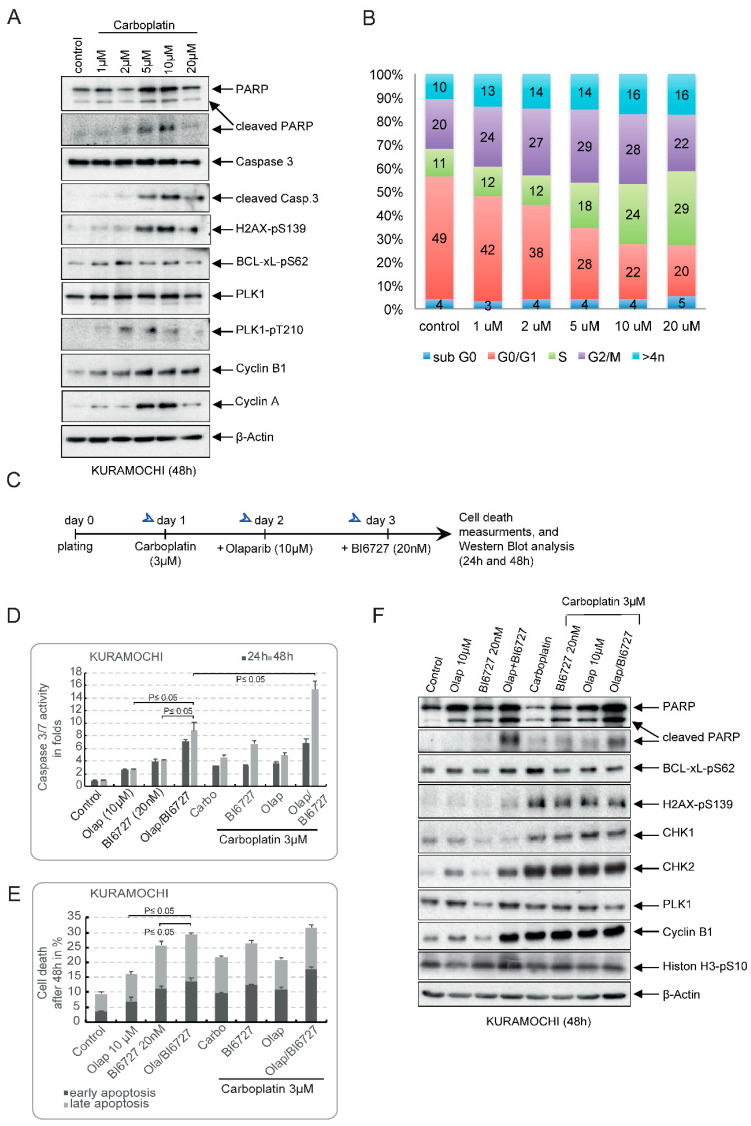
PLK1i and PARPi mediate sensitivity to HR-deficient HGSOC with KRAS amplification to Carboplatin. KURAMOCHI cells were treated with increasing concentrations of Carboplatin, 1 µM to 20 µM. Cells were harvested after 48 h. Cell lysates were prepared for Western blot using the indicated antibodies (**A**), and the cell cycle distribution of treated cells was analyzed using FACS (**B**). (**C**) Treatment schedule: KURAMOCHI cells were treated with Carboplatin (3 µM) on day 1. Following this, cells were sequentially treated with Olaparib (10 µM) on day 2 and BI6727 (20 nM) on day 3. Cells were harvested 24 h (day 4) and 48 h (day 5) after completion of the combinatorial treatments, and further experiments were carried out. (**D**,**E**) Apoptosis was first assessed by measuring Caspase 3/7 activity in cell lysates of cells incubated with the different single or combinatorial treatments and by measuring cell death after 24 h and 48 h using Annexin V/AAD. The results are presented as mean ± SD. (*n* = 3, *p* ≤ 0.05). (**F**) Cell lysates of KURAMOCHI cells treated with single agents or combinations, as in (**C**), were prepared for Western blot using the indicated antibodies.

Carboplatin belongs to the standard treatment regimen administered to patients with ovarian cancer. To test the single effect of Carboplatin on OVSAHO and KURAMOCHI, we incubated both cell lines for 48 h with increasing concentrations ranging from 1 µM up to 20 µM. Western blot analysis showed that concentrations ≥5 µM triggered an apoptotic response in KURAMOCHI cells, as indicated by increased PARP cleavage and an uptake in Caspase 3 cleavage (Figure 5A). We noticed that, in contrast to low concentrations of Carboplatin, which barely induce DSB (faint γ-H2AX staining at 1 µM and 2 µM), higher concentrations increased γ-H2AX staining (Figure 5A). Furthermore, we observed a slight Carboplatin concentration-dependent increase in G2-M cells, as indicated in Figure 5B, and by the accumulation of Cyclin A and Cyclin B1 in Figure 5A. Similar results were obtained with the OVSAHO cell line, except for the incidence of γ-H2AX foci, which already occurred at 1 µM Carboplatin (Supplementary Figure S6A). This is again suggestive of the low sensitivity of KURAMOCHI cells to DNA-damaging agents (Figure 5A,B). Based on these findings, we used 3 µM Carboplatin for our following combinatorial treatments.

We next examined whether the coadministration of PLK1i and PARPi enhances the apoptotic response to Carboplatin. To this end, we incubated KURAMOCHI and OVSAHO cells first with 3 µM Carboplatin on day 1, then sequentially we added Olaparib on day 2 and BI6727 on day 3 (Figure 5C and Figure S6C). Cells were harvested after 24 h and 48 h, and apoptosis was measured by Caspase 3/7 activation assays and Annexin V/7-AAD assay. For comparison, we again performed single and double treatments of BI6727 and Olaparib, the efficacy of which was compared to that of the triple combination (Figure 5D–F and Figure S6D–F). We were able to confirm that sequential Olap/BI6727 treatment significantly enhances the apoptotic response compared to a single treatment in KURAMOCHI. We observed a significant 8.9-fold increase in Caspase 3/7 activation (Figure 5D) and 28% cell death in Annexin V/AAD assay (Figure 5E) after 48 h. Regarding the Carboplatin combinations, again, the sequential combination Carboplatin/Ola/BI6727 triggered the highest apoptotic response after 48 h with a 15-fold activation of Caspase 3/7 compared to only 4.9-fold and 6.8-fold, respectively, for the double combinations Carbo/Olap, Carbo/BI6727 and 4.4-fold in the case of single carboplatin treatment (Figure 5D). Furthermore, the Annexin V/AAD assay confirmed that Carbo/Ola/BI6727 is the most effective treatment compared to double combinations or single Carboplatin (Figure 5E). In line with these results, Western blot analysis showed that the most substantial PARP cleavage, indicating apoptosis, was seen in the Olap/BI6727 and Carbo/Olap/BI6727 treatment groups (Figure 5F).

Interestingly, levels of BCL-XL-pS62, an apoptotic marker, were more elevated in the Olap/BI6727 combination compared to Carbo/Olap/BI6727 (Figure 5F and Figure S6F). Considering that serine 62 is a critical site for BCL-XL response to microtubule damage, we suggest that the mechanisms underlying the apoptotic stimuli elicited by Olap/BI6727 and the Carbo/Olap/BI6727 combinations may differ from each other, with the former targeting interphase and mitotic microtubules and the triple combination primarily triggering a DNA damage response. Comparable results were obtained with the OVSAHO cell line (Supplementary Figure S6D–F). 

In summary, our results suggest that PLK1i and PARPi potentiate the effect of Carboplatin on HR-deficient HGSOC cells, particularly those harboring KRAS amplification.

### 2.6. Inhibition of PLK1 and PARP Increases Carboplatin Response in Ovarian Patient-Derived Spheroid Cultures

**Figure 6:**
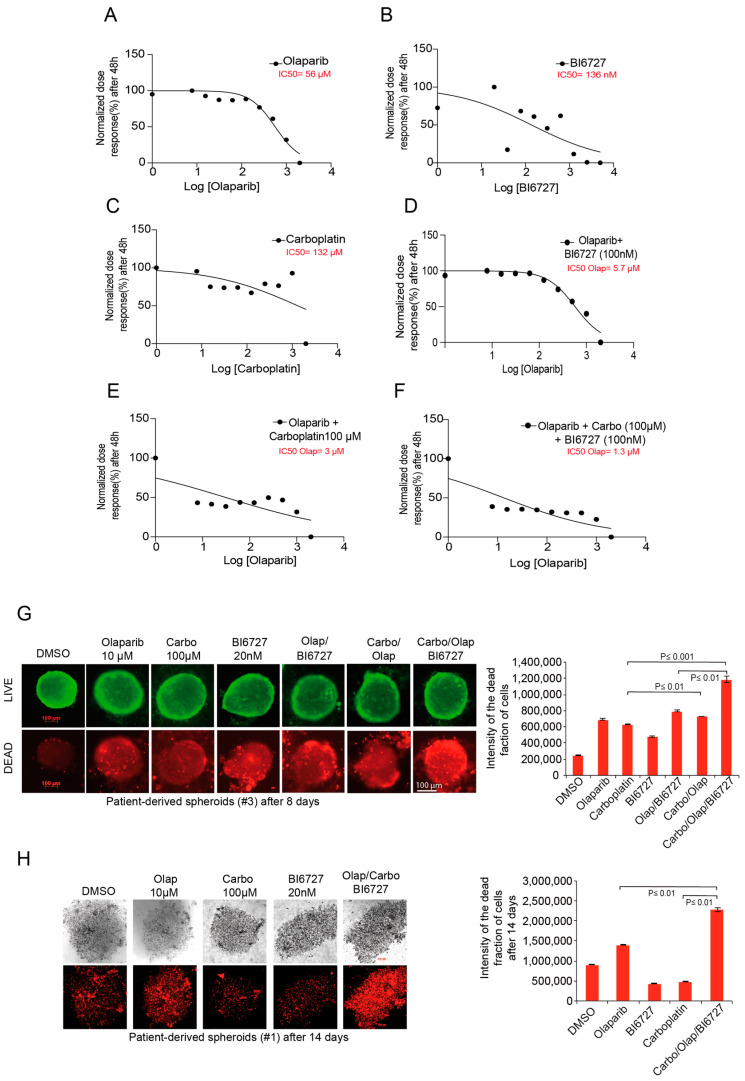
PARPi and PLK1i combination strongly enhances apoptosis-dependent death of patients-derived tumor cells. (**A**–**C**) Primary tumor cells of patients were treated with increasing concentrations of Olaparib, BI6727, and Carboplatin as single agents or combinations (**D**–**F**). The cell viability was determined after 48 h, and the IC_50_ of the different agents was calculated. (**G**,**H**) 3D cultures grown from tumor cells of patients were treated with 10 µM Olaparib, 20 nM BI6727, and 100 µM Carboplatin as single or combinatorial treatments for 8 days. Cells were stained, and the fraction of dead cells was quantified using immunofluorescence. The results are presented as mean ± SD and statistically analyzed (*n* = 5, *p* ≤ 0.001, *p* ≤ 0.01).

To study the relevance of our triple combination for cancer patient treatment, we analyzed ovarian cancer cells isolated from tumors of relapsed and newly diagnosed patients just after debulking surgery prior to chemotherapy. First, we evaluated the single effect of Olaparib, BI6727, and Carboplatin on the patient-derived tumor cells. The IC_50_ value for the single treatment was 56 µM, 136 nM, and 132 µM, respectively, for Olaparib, BI6727, and Carboplatin (Figure 6A–C). Moreover, the pretreatment of tumor cells with 100 nM BI6727 reduced the IC_50_ of Olaparib by 10-fold to 5.7 µM (Figure 6D), indicating that, similar to previous cancer cell lines, PLK1 inhibition strongly sensitizes patient-derived tumor cells to Olaparib. More importantly, we found that treating patients with 100 µM carboplatin further reduced the IC_50_ of Olaparib to 3 µM, and 100 µM carboplatin combined with 100 nM BI6727 reduced the IC_50_ of Olaparib to a low 1.3 µM (Figure 6E,F).

Next, we investigated whether blocking PLK1 and PARP would enhance Carboplatin response of 3D-primary spheroid cultures derived from two different patients diagnosed with ovarian cancer. Therefore, we tested the combination of 100 µM Carboplatin, 10 µM Olaparib, and 100 nM BI6727 using the same sequential treatment as described in Figure 5C. Life/death assays performed after eight days in differently treated spheroid cultures confirmed that in both patients the Carbo/Olap/BI6727 combination resulted in the most significant increase in the fraction of dead cells compared with all individual treatments or double combinations (Figure 6G,H). Together, the patient tumor-derived 3D spheroid assays support the clinical significance of the combination of PLK1i and PARPi with Carboplatin.

## 3. Discussion

Many PARP inhibitors have been developed and approved by the FDA for treating patients with recurrent Platinum-sensitive ovarian cancer, with or without the loss of the BRCA1/2 gene [15,55,56,57]. Unfortunately, tumors adapt to PARPi-induced stress despite an initial positive response, and patients rapidly develop resistance [42]. Several potential mechanisms for PARPi resistance have been reported [58,59]. Interestingly, a recent study has linked the acquired resistance to PARPi to an upregulated RAS-RAF-MEK pathway and proposed using MEKi to sensitize cancers showing RAS overactivation to PARP inhibitors [42,59]. Intriguingly, KRAS amplification was found in 13.2% of type II ovarian cancer tissue samples and might represent an essential factor in the growth and resistance of HGSOC [5]. Consistent with this, our initial gene expression analysis using the KM plotter tool pointed out that KRAS overexpression significantly reduced OS in a cohort of stage III HGSOC patients who had previously received Platinum-based chemotherapy (Supplementary Figure S1A). Considering these findings, we sought to explore the potential role of KRAS overexpression in the resistance of HR- deficient HGSOC, first to PARPi, and then to Platine-based standard therapy. To do this, we selected two BRCA2-defective HGSOC cell lines with different KRAS status. Unlike the OVSAHO cell line, KURAMOCHI cells are reported to have KRAS gene amplification [45,46]. We were able to show that this cell line displays RAS-RAF-MEK-ERK pathway overactivation, as indicated by elevated levels of p-ERK1/2 (Supplementary Figure S1D). Furthermore, our experiments confirmed the significantly reduced sensitivity of KURAMOCHI to single PARPi, even at high concentrations (Figure 1). Owing to the fact that the second BRCA2-deficient cell line, OVSAHO, responded well to Olaparib treatment, it is reasonable to assume that KRAS amplification and concomitant increased activation of the RAS-RAF-MEK-ERK pathway may be factors playing a role in KURAMOCHI resistance to PARPi.

ERK1/2 kinase is cytoplasmic under unstimulated conditions. Following activation, p-ERK1/2 translocates to the nucleus, where it regulates the activity of many transcriptional factors, such as c-Jun, c-Fos, and c-Myc, promoting cell proliferation, survival, and mobility [60]. The complete activation of ERK1/2 relies on the phosphorylation of two residues within the TEY motif. In contrast, the termination of the signal depends on the activity of the dual-specificity phosphatase “mitogen-activated protein kinase phosphatase-1” (MKP-1) [61]. Furthermore, MKP-1 expression in the tumor tissues of ovarian cancer patients with stage III/IV disease is significantly lower than in patients with stage I/II [60], and a negative correlation between p-ERK1/2 and MKP-1 expressions has been be established [62]. In addition, ERK1/2 kinase promotes its activation by triggering the proteasomal degradation of MKP-1 [63]. Based on our data and the findings reported in the aforementioned studies, it is highly likely that an enhanced survival pathway and repressed apoptosis due to uncontrolled activation of ERK1/2 in KURAMOCHI cells counteracts the PARPi effect. Nevertheless, two aspects require further exploration: (i) whether the increased levels of p-ERK1/2 can be directly correlated with KRAS amplification, and (ii) clarification of the role of MKP-1 in the context of the PARPi resistance of HGSOC with KRAS amplification.

Synthetic lethal drug screening reported that KRAS-mutated cancer cells seem vulnerable to PLK1 inhibition [64] and depend more heavily on PLK1 activity for mitotic progression [65]. Hence, we wondered whether KRAS amplification, as for its oncogenic mutations, would render HR-deficient HGSOCs sensitive to PLK1i.

Indeed, we found that the response to PLK1i differed depending on the KRAS status of both cell lines. Single BI6727 treatment was highly efficacious in triggering cell death in the KRAS-amplified KURAMOCHI cell line, while generating a modest response in the OVSAHO cell line (Supplementary Figure S3). Furthermore, we observed that PLK1i causes a decrease in cells arrested in the G2/M phase, as well as a significant increase in the sub-G1 fraction in KURAMOCHI cells (Supplementary Figure S3E,G). This increase, however, is seen only after 48 h of treatment and with high concentrations of BI6727 (50 nM and 100 nM). We have previously demonstrated that inhibiting PLK1 causes cell arrest in mitosis, specifically in prophase, thereby preventing mitosis from resuming [34,66]. If this arrest is maintained for an extended period, in this case, 48 h, it triggers a cell death program known as “mitotic catastrophe”, in which the cells undergo apoptosis. As KURAMOCHI cells seem to be sensitive to PLK1 inhibition, the use of high BI6727 concentrations (50 nM and 100 nM) triggered significant apoptosis within the G2/M fraction of cells, which was translated into a reduction of the G2/M proportion of cells and an increase in the sub-G1 apoptotic fraction seen in the FACS measurement of the cell cycle (Supplementary Figure S3E,G).

Moreover, our data demonstrate that regardless of the treatment sequence, PLK1 inhibition similarly boosts the cellular apoptotic response to PARPi and reduces the potential of both BRCA2-deficient HGSOCs to form 2D colonies over the long term (Figure 2 and Figure 3). However, the KURAMOCHI cell line responds much better to the treatment sequence in which PLK1 is inactivated first, followed by PARPi. This is an intriguing observation, as KRAS amplification seems to render KURAMOCHI cells more sensitive to PARP inhibition when cells are already arrested in mitotic prophase following initial BI6727 treatment. This was particularly clear after 48 h treatment, where the BI6727/Olaparib combination strongly reduced PLK1 protein levels, indicating the increased apoptosis taking place in mitotic cells (Figure 2G). This suggests the existence of a critical interaction between PARP and PLK1, which is potentially involved in regulating cell division, particularly in triggering mitotic catastrophe, and is consistent with the previously reported requirement of PARP in mitotic spindle assembly and structure [67].

The role of PLK1 in DNA damage response and repair is well established [22,23,68], and its activity is indispensable for the cells to enter mitosis upon recovery from the DNA damage-induced G2 arrest [48]. Mechanistically, blocking PARP enzymatic activity upon DNA damage triggers the conversion of ssDNA breaks to lethal DSBs [69]. Thus, the combination of PLKi and PARPi presumably results in inhibiting the DNA damage response and repair, consequently retaining cells in the G2-phase by blocking the “G2/M” transition upon damage recovery. Furthermore, because PARP is involved in other DNA damage repair mechanisms, such as BER, NHEJ, and alternative-NHEJ [70], both HR-deficient and proficient cells may benefit from the combination of PLK1i and PARPi upon DNA damage induction. Indeed, our experiments revealed that the MMS/Ola/BI6727 combination, independent of KRAS status, increased the frequency and intensity of unrepaired γ-H2AX foci. Furthermore, these DSB foci were persistent over time, promoting cell death and significantly reducing long-term cell survival compared to single and double combinations (Figure 4 and Figure S5).

Platinum-based chemotherapy is the current standard of care for ovarian cancer patients [71]. However, we found that KRAS amplification can promote resistance to single carboplatin treatment and even to the Carboplatin/PARPi combination, as observed in KURAMOCHI cells (Figure 5). By adding BI6727 to the Carbo/Olaparib combination, we were able to potentiate the effect of Carboplatin on OVSAHO cells remarkably. However, and more importantly, PLK1i restored the sensitivity of the KRAS-amplified KURAMOCHI cell line to Carboplatin in the triple Carbo/Ola/BI6727 combination, resulting in a substantial increase in apoptosis and cell death after treatment (Figure 5 and Figure S6).

The potential of the combination of Carboplatin, PARPi, and PLK1i was demonstrated in 3D spheroid assays of primary ovarian cancer cells originating from two different patients. Our experiments showed that the triple combination Carbo/Olap/BI6727 significantly enhanced apoptosis-dependent cell death compared to the respective single or double treatments (Figure 6).

In summary, we can say that our study addressed three significant aspects that could have a clinical and therapeutical impact on HGSOC patients with defective HR repair pathway and KRAS amplification:

(1) It was shown that resistance to PARP inhibition in HR-deficient HGSOC with amplified KRAS gene and overactivated RAS-RAF-MEK-ERK pathway could be counteracted by sequential inhibition of PARP and PLK1 kinase.
(2) We showed that the resistance of HR-deficient HGSOC cells with KRAS amplification to standard Platinum-based therapy can be reversed by combining sequential PLK1i and PARPi with Carboplatin.
(3) Finally, KRAS amplification could serve as a predictive marker for the efficacy of PLK1i-based combination therapy in HR-deficient ovarian cancer patients.

## 4. Material and Methods

### 4.1. Cell Culture

The BRCA2-deficient HGSOC cell lines KURAMOCHI and OVSAHO were a gift from the lab of Dr. David Bowtell Peter (MacCallum Cancer Centre Victorian Comprehensive Cancer Centre, Melbourne, Australia). The cells were cultured in RPMI 1640 (Gibco, Thermo Fisher Scientific, Waltham, MA, USA), containing 10% FCS (Gibco, Thermo Fisher Scientific, Waltham, MA, USA) and 1% Penicillin/Streptomycin (Sigma-Aldrich, St. Louis, MO, USA). Primary cells were isolated from ovarian cancer tissues using a tumor dissociation kit (Max Miltenyi 130-095-929) and a tumor cell isolation kit (Max Miltenyi 130-108-339) following the manufacturer’s instructions. 

### 4.2. Patients and Samples (Primary Cell Culture)

This study was conducted according to the “REporting recommendations for tumor MARKer prognostic studies” [72]. To establish primary patient-derived ovarian cancer cell cultures, we analyzed samples from patients undergoing surgical resection between January 2015 and March 2018 at the Department of Gynecology of the Goethe University Hospital in Frankfurt am Main, Germany. Sufficient archival material for immunohistochemical analysis was available for the samples with validated diagnoses. The Local Research Ethics Committees approved the human tissue studies, and all samples were processed anonymously.

### 4.3. Colony Formation Assay

Two thousand cells were seeded in six-well plates. Cells were treated with Olaparib (10 µM), and BI6727 (20 nM) as single treatments or in combination. The cells were first treated with MMS, Olaparib, and BI6727 as single agents or in combination. After 3 weeks of incubation, the colonies were fixed using 70% EtOH and stained with Coomassie Brilliant Blue. Colony images were taken using AxioObserver Z1 microscope (Zeiss) and the ChemiDoc MP system (BioRad, Hercules, CA, USA). The numbers of grown colonies were counted using the automated counting plugin of Image J.

### 4.4. Three-Dimensional (3D) Cultures

A cell suspension of 1000 cells/50 μL was prepared and pipetted from the topside into a 96-well Perfect 3D Hanging Drop plate (BioTrend, Köln, Germany). Plates were incubated at 37 °C for one day until hanging drops had developed. The 3D culture was harvested on a 96-well plate covered with 1% agarose by low-spin centrifugation. Treatment of cells with single and combinatorial treatments was performed as indicated. Cells were stained with the LIVE/DEAD viability/cytotoxicity kit (Molecular Probes/Thermofisher, Waltham, MA, USA) for 30 min and inspected using a fluorescence microscope. While the polyanionic dye calcein is retained in live cells, producing an intense uniform green fluorescence, EthD-1 enters cells with damaged membranes, producing a bright red fluorescence upon binding to nucleic acids in dead cells. The ratios of viable/dead cells were calculated with the software ImageJ Fiji.

### 4.5. Cell Cycle Analysis and Apoptosis Assay

Treatment with MMS, Carboplatin, BI6727, and Olaparib as single agents or in combination was conducted for 48 h. Cell cycle measurements were performed after predefined time intervals. Cells were harvested, washed, fixed with 70% EtOH, and stained as described [73]. Cell cycle quantification was performed using FACS Calibur and Cellquest Pro software (both BD Biosciences, Franklin Lakes, NJ, USA). The sub-G1 indicates the apoptotic fraction of cells resulting from the different treatments. The activity of Caspase-3/7 was determined using the Caspase-Glo 3/7 Assay (Promega, Madison, WI, USA). Twenty microliters of substrate per well were applied, and after 30-min shaking at room temperature in the dark, luminescence was detected (Victor X4, Perkin Elmer, Waltham, MA, USA). Data analysis was performed online by a network analyst and GraphPad Prism.

### 4.6. Genotoxic Treatment

Methyl methanesulfonate (MMS) (Sigma) was diluted in sterile water to a concentration of 1 M and added to the cell culture medium to a final concentration of 0.5 mM. Cells were treated for 1 h. After that, the medium was replaced either by a fresh medium or medium containing the inhibitors as indicated. Cells were further incubated for 24 h.

### 4.7. Cell Cycle-Specific Analysis of DSB Repair

For specific analysis of DSB repair at the G2 phase, the thymidine analog EdU (10 mM, EdU click-647, # 7777.1, Roth) was added to growing cells 1 h prior to MMS (0.5 mM) treatment and maintained throughout the repair incubation period. After fixation, cells were immunostained with γ-H2AX, DAPI, and EdU. EdU-positive cells were identified as S-phase cells, while EdU-negative cells were categorized as either G1 or G2 based on their DNA content. γ-H2AX foci were counted exclusively in the G2 phase using ImageJ software.

### 4.8. Immunofluorescence Assays

Cells were grown on a glass coverslip, fixed, and permeabilized with 4%PFA/0.5%-Triton X-100 for 20 min at room temperature, then washed with PBS before adding appropriate primary and secondary antibodies. Cells were stained with the following primary antibodies: Rabbit γ-H2AX (cell signaling, # 9718) and the secondary antibodies: FITC-conjugated donkey anti-rabbit (Jackson Immunoresearch, West Grove, PA, USA). DNA was stained using DAPI (4′,6-diamidino-2-phenylindol-dihydrochloride) (Roche, Mannheim, Germany). Slides were examined using an Axio Imager 7.1 microscope (Zeiss, Göttingen, Germany), and images were taken using an Axio Cam MRm camera (Zeiss, Göttingen, Germany).

### 4.9. Western Blot Analysis

Protein extracts of cells were prepared by lysis in RIPA buffer (Sigma) supplemented with protease inhibitor (Complete protease inhibitor cocktail, Roche). Protein extracts (25 μg) were separated by SDS-PAGE and transferred onto membranes using the TransBlot Turbo Transfer System (BioRad). After blocking with 5% BSA in PBS with 0.1% Tween-20 for 30 min, the membrane was incubated with primary antibodies for 1 h at room temperature. HRP-linked secondary antibodies were incubated 30 min at room temperature followed by ECL detection (ECL Chemiluminescent Western Blot Substrate, Pierce, Thermo Fisher Scientific, Waltham, MA, USA).

### 4.10. Antibodies for Western Blot and Chemicals

Primary antibodies were obtained from the following sources:

PLK1 (05-844), phospho-Histone H3 (Ser10) (05806) from Millipore, Cyclin B1 (GNS1), CyclinA (B-8) from Santa Cruz Biotech. PARP (9542), cleaved PARP (9542), Caspase-3 (9668), and cleaved Caspase-3 (8610) from Cell signaling. BCL-XL pS62 (Abcam # ab30655), γ-H2AX (cell signaling, # 9718), CHK1 (2GD15) (cell signaling # 2360), CHK2 (D9C6) (cell signaling # 6334) and β-Actin (A2228-100UL) from Sigma-Aldrich served as a loading control. Secondary antibodies for Western blot analysis against rabbit (NA934V) and mouse (NXA931) IgG were obtained from GE Healthcare. 

Reagents were purchased from the following sources: 

Carboplatin (# 41575-94-4, Sigma Aldrich), BI6727 (BYT-ORB181049), Selleckchem, Propidium iodide (440300250), Acros Organics, RNAse A (1007885) Qiagen, PE Annexin V (556421) and 7AAD (21-68981E) BD Biosciences, Olaparib (#7579, Tocris, Wiesbaden, Germany), and Methyl methansulfonat (MMS) (# 66-27-3, Sigma-Aldrich).

### 4.11. Statistical Analysis

All experiments were performed at least three times and are displayed as mean and standard error of the mean. Statistical significance was assessed by Student’s *t*-test (two-tailed and paired) using Excel 2010 (Microsoft) as well as GraphPad Prism 7 (GraphPad, La Jolla, CA, USA). Significant differences (* *p* ≤ 0.05; ** *p* ≤ 0.01; *** *p* ≤ 0.001) are indicated in the figures with asterisks.

## Data Availability

The data and material that support the findings of this study are available within this article and from the corresponding authors upon reasonable request.

## Supplementary Materials

The following supporting information can be downloaded at: https://www.mdpi.com/article/10.3390/ijms231810892/s1.
