# Supplementary material for: Sequential Targeting of PLK1 and PARP1 Reverses the Resistance to PARP Inhibitors and Enhances Platin-Based Chemotherapy in BRCA-Deficient High-Grade Serous Ovarian Cancer with KRAS Amplification
Source: Int J Mol Sci. 2022 Sep 17;23(18):10892. doi: 10.3390/ijms231810892 (PMC9502276; doi:10.3390/ijms231810892)
Supplement: Supplementary file 1 [file ijms-23-10892-s001.zip › Supplementary Figures 1-4.pdf]

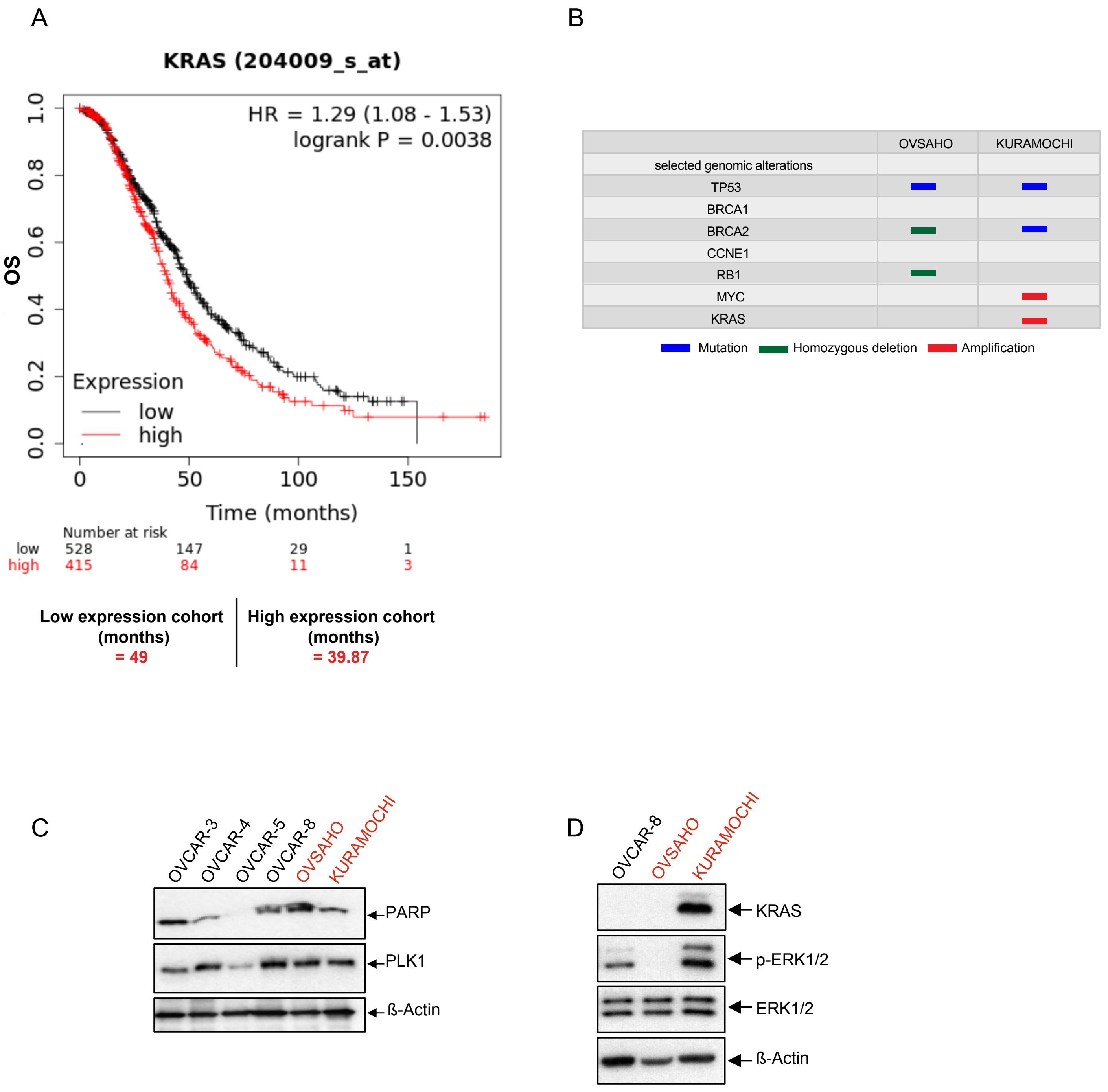

Supplemental Figure S1

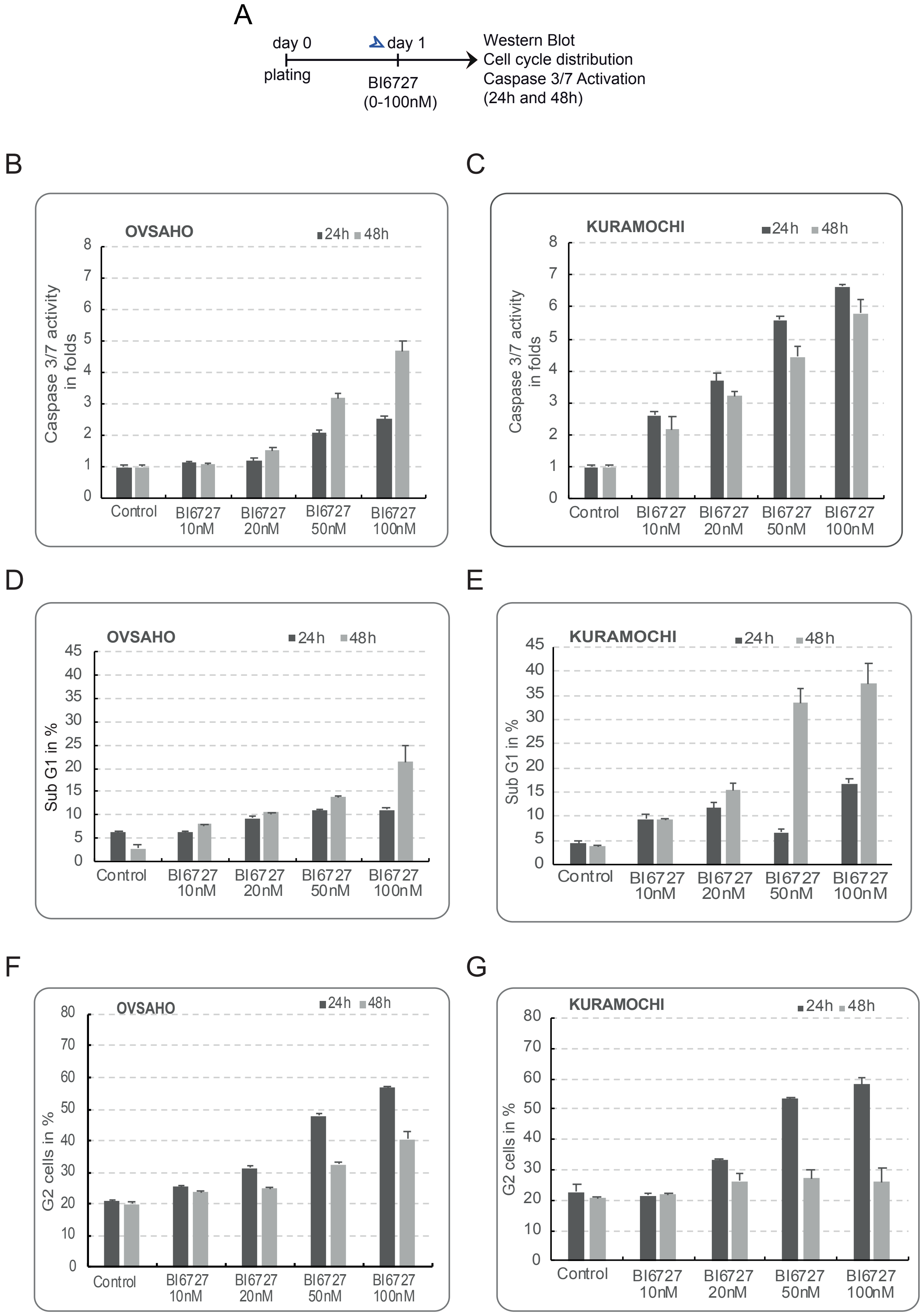

Supplemental Figure S2

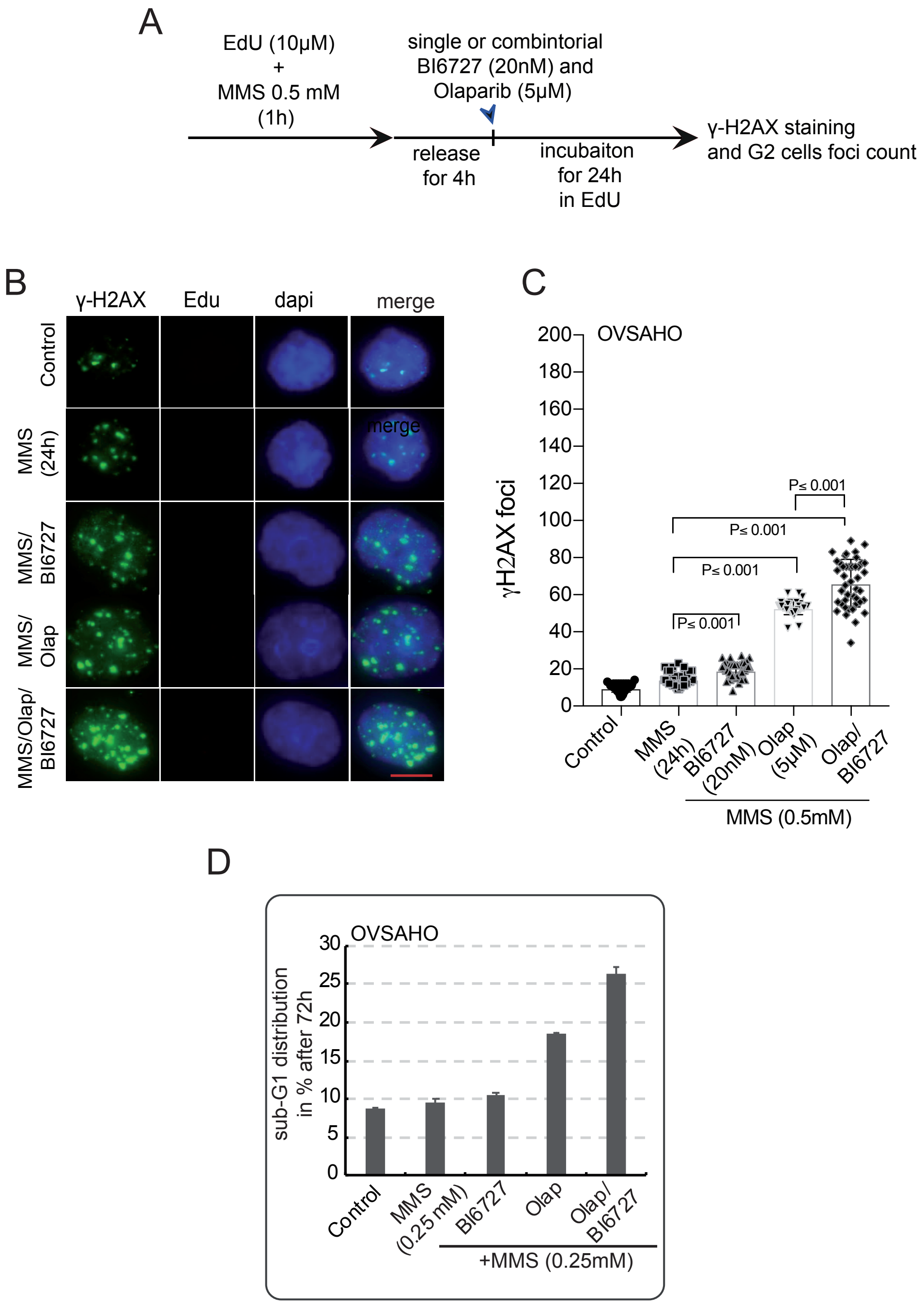

Supplemental Figure S3

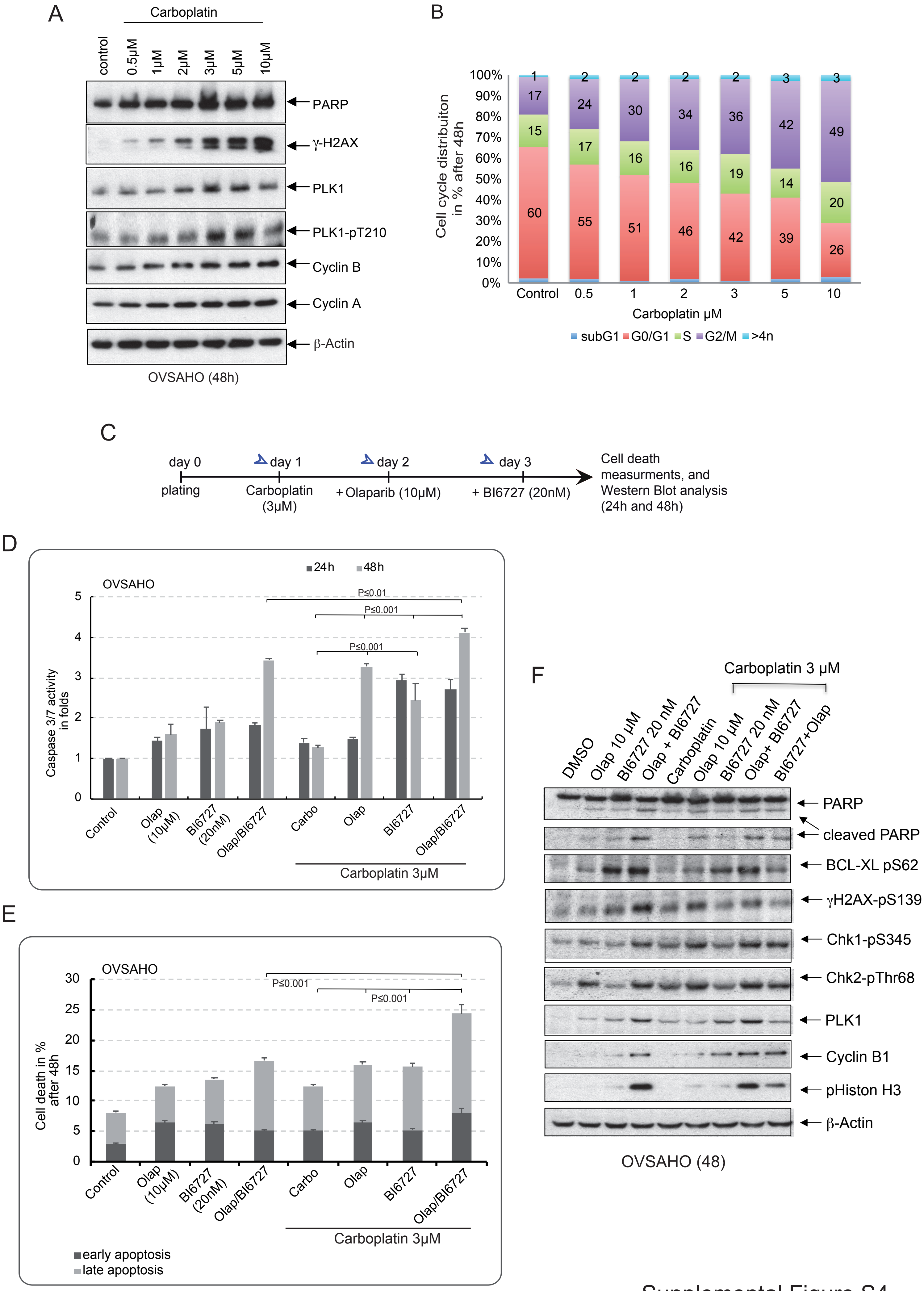

Supplemental Figure S4
