# Supplementary material for: Sequential Targeting of PLK1 and PARP1 Reverses the Resistance to PARP Inhibitors and Enhances Platin-Based Chemotherapy in BRCA-Deficient High-Grade Serous Ovarian Cancer with KRAS Amplification
Source: Int J Mol Sci. 2022 Sep 17;23(18):10892. doi: 10.3390/ijms231810892 (PMC9502276; doi:10.3390/ijms231810892)
Supplement: Supplementary file 1 [file ijms-23-10892-s001.zip › Supplementary Materials.pdf]

Sequential targeting of PLK1 and PARP1 reverses the resistance to PARP inhibitors and enhances Platin-based chemotherapy in BRCA-deficient high-grade serous ovarian cancer with KRAS amplification.

Supplementary Materials

Supplementary Figures:

Supplemental Figure S1. Relationship between KRAS amplification and overall survival in patients who received primary cytoreductive surgery followed by standard platinum chemotherapy.

(A) Kaplan-Meier survival analysis showed that KRAS amplification was associated with shorter overall survival (OS) (Log-rank test, P = 0.0038), **(B)** The mutational profile of the two HGSOC cell lines KURAMOCHI and OVSAH were identified from the literature [46]. **(C)** Western blot of different ovarian cancer cells showing the levels of PLK1, PARP-1 and ß-actin**. (D)** Western blot of ovarian cancer cells showing high protein levels of KRAS, p-ERK1/2 in KURAMOCHI cells.

Supplemental Figure S2. KRAS amplification mediates sensitivity to PLK1i in HGSOC cells with BRCA2 deficiency. A- Treatment schedule: KURAMOCHI and OVSAHO cells were treated with increasing concentrations (10 - 100 nM) of the PLK1 inhibitor BI6727. Cells were harvested after 24 h and 48 h, and further experiments were carried out. **(B-C)** Apoptosis was assessed at the indicated time points by measuring Caspase 3/7 activity in the lysates of cells. The results are presented as mean ± SD. (n=3). **(D-E)** Cell death was also assessed by quantifying the sub-G1 phase. The results are presented as mean ± SD. (n=3). **(F-G)** The cell cycle distribution of treated KURAMOCHI and OVSAHO was analyzed 24 h and 48 h post-BI6727 treatment using FACS. The resulting G2 fractions of both cell lines are displayed. The results are presented as mean ± SD (n=3).

Supplemental Figure S3. Combining PLK1i and PARPi enhances the DNA damage effect of MMS and reduces the viability of BRCA2-deficient HGSOC. A- Treatment schedule: OVSAHO cells were EdU labeled and puls-treated with MMS (0.5 mM) for 1 h. The cells were released in EdU containing medium for 4h, then treated with the single agents or combined for 24h in the presence of EdU. γ-H2AX foci were analyzed in EdU-negative G2-phase cells. **(B)** IF images show OVSAHO cells with γ-H2AX foci and negative EdU staining at 24 h post-treatments. **(C)** Quantification of γ-H2AX foci 24 h post-treatment. The results are presented as mean ± SEM (n= 50 cells per treatment, *** P≤ 0.001). **(E)** Cell death was assessed in treated OVSAHO cells as in (A) by quantifying the sub-G1 phase after 72 h. The results are presented as mean ± SD. (n=3, *** P≤ 0.001, ** P≤ 0.01).

Supplemental Figure S4. PLK1i and PARPi boosts the sensitivity of BRCA2-deficient HGSOC to Carboplatin. OVSAHO cells were treated with increasing concentrations of Carboplatin, 0.5 µM to 10 µM. Cells were harvested after 48 h. Cell lysates were prepared for Western Blot using the indicated

antibodies **(A),** and the cell cycle distribution of treated cells was analyzed using FACS **(B).** **(C)** Treatment schedule: OVSAHO cells were treated with Carboplatin (3 µM) on day1. Following this, cells were sequentially treated with Olaparib (10 µM) on day 2 and BI6727 (20 nM) on day3. Cells were harvested 24 h and 48 h after completion of the combinatorial treatments, and further experiments were carried out. **(D-E)** Apoptosis was first assessed by measuring Caspase 3/7 activity in cell lysates of cells incubated with the different single and combinatorial treatments and by measuring cell death after 24 h and 48 h using Annexin V/ AAD. The results are presented as mean ± SD (n=3, * P≤ 0.05). **(F)** Cell lysates of OVSAHO cells treated with single agents and combinations, as in (C), were prepared for Western blot using the indicated antibodies.
